# The Ultrastructure of Tissue Damage by Amyloid Fibrils

**DOI:** 10.3390/molecules26154611

**Published:** 2021-07-29

**Authors:** Haruki Koike, Masahisa Katsuno

**Affiliations:** Department of Neurology, Graduate School of Medicine, Nagoya University, Nagoya 466-8550, Japan; ka2no@med.nagoya-u.ac.jp

**Keywords:** chemotherapy, diflunisal, electron microscopy, inotersen, neurodegeneration, pathogenesis, pathology, patisiran, plasma cell dyscrasia, tafamidis

## Abstract

Amyloidosis is a group of diseases that includes Alzheimer’s disease, prion diseases, transthyretin (ATTR) amyloidosis, and immunoglobulin light chain (AL) amyloidosis. The mechanism of organ dysfunction resulting from amyloidosis has been a topic of debate. This review focuses on the ultrastructure of tissue damage resulting from amyloid deposition and therapeutic insights based on the pathophysiology of amyloidosis. Studies of nerve biopsy or cardiac autopsy specimens from patients with ATTR and AL amyloidoses show atrophy of cells near amyloid fibril aggregates. In addition to the stress or toxicity attributable to amyloid fibrils themselves, the toxicity of non-fibrillar states of amyloidogenic proteins, particularly oligomers, may also participate in the mechanisms of tissue damage. The obscuration of the basement and cytoplasmic membranes of cells near amyloid fibrils attributable to an affinity of components constituting these membranes to those of amyloid fibrils may also play an important role in tissue damage. Possible major therapeutic strategies based on pathophysiology of amyloidosis consist of the following: (1) reducing or preventing the production of causative proteins; (2) preventing the causative proteins from participating in the process of amyloid fibril formation; and/or (3) eliminating already-deposited amyloid fibrils. As the development of novel disease-modifying therapies such as short interfering RNA, antisense oligonucleotide, and monoclonal antibodies is remarkable, early diagnosis and appropriate selection of treatment is becoming more and more important for patients with amyloidosis.

## 1. Introduction

Amyloidosis is a group of diseases in which amyloid fibrils are deposited in tissues. According to a report from the International Society of Amyloidosis nomenclature committee in 2020, 36 proteins are listed as precursors of amyloid fibrils that may be deposited in the extracellular spaces of tissues of various organs [1]. About half of these proteins are associated with localized amyloidosis, i.e., confined to a specific single organ, as is the case of Alzheimer’s disease, wherein amyloid deposition occurs only in the central nervous system [1]. The other types of amyloidosis are of the systemic type, although the light and heavy chains of immunoglobulins among the proteins in these types may also cause localized amyloidosis [2]. Transthyretin (TTR) and the immunoglobulin light chain (AL) are the two major proteins that cause systemic amyloidosis, namely ATTR amyloidosis and AL amyloidosis, respectively [3]. In these diseases, amyloid deposition occurs in various organs, including the heart, lungs, liver, kidneys, gastrointestinal tract, soft tissues, and peripheral nervous system, resulting in multi-organ failure [4,5,6]. The mechanism of organ dysfunction resulting from amyloidosis has been a topic of debate. The restriction of ventricular wall movement resulting from massive amyloid deposition has been considered as the cause of heart failure in patients with cardiac amyloidosis [7,8]. Meanwhile, electron microscopic studies have demonstrated the degeneration of tissues surrounding amyloid deposits, suggesting that amyloid fibrils or non-fibrillar precursors exert deleterious effects on cells [9].

In this review, we describe the ultrastructure of tissue damage resulting from amyloid deposition by focusing on two major systemic amyloidoses, i.e., ATTR amyloidosis and AL amyloidosis. Because novel disease-modifying therapies for ATTR and AL amyloidoses appear one after another, therapeutic insights based on the pathophysiology of amyloidosis are also described. This article is based on previously conducted studies and does not contain any studies with human participants or animals performed by any of the authors.

## 2. What Are ATTR Amyloidosis and AL Amyloidosis?

ATTR amyloidosis and AL amyloidosis are major systemic amyloidoses that result in a fatal clinical outcome, particularly due to heart failure [10,11]. ATTR amyloidosis is caused by the deposition of amyloid fibrils composed of TTR, which is produced in the liver and physiologically functions as a transporter of thyroxine and retinol-binding protein [9]. This disease mainly consists of hereditary ATTR (ATTRv; v stands for variant) amyloidosis, alternatively known as familial amyloid polyneuropathy, and wild-type ATTR (ATTRwt) amyloidosis, also known as senile cardiac or systemic amyloidosis, based on the presence or absence of a mutation in *TTR* [6]. ATTRv amyloidosis was regarded as a disease restricted to specific areas of Portugal [12], Japan [13], and Sweden [14], while ATTRwt amyloidosis was considered a type of cardiomyopathy found in the elderly [15,16,17]. The advances in the diagnostic techniques and increased recognition of these diseases led to an increase in the number of newly diagnosed patients throughout the world [18]. In contrast, AL amyloidosis is caused by the deposition of amyloid fibrils composed of immunoglobulin light chains that are produced by clonal plasma or B cells [5]. AL amyloidosis may be localized due to in situ production of light chains, resulting in a benign clinical course [2]. However, systemic deposition leads to severe multiple organ dysfunction, including neuropathy, cardiomyopathy, and nephrotic syndrome [19].

Well-known textbook features of polyneuropathies resulting from ATTR and AL amyloidoses are progressive symmetrical sensory impairments collectively known as dissociated sensory loss, which is characterized by a loss of nociception and thermal sensation, as well as autonomic dysfunction such as diarrhea/constipation, orthostatic intolerance, dysuria, and erectile dysfunction, due to the loss of small-diameter nerve fibers [4,20,21]. These characteristics are particularly conspicuous during the early phase of neuropathy, and motor dysfunction is considered a later manifestation [11]. However, some patients, particularly those with late-onset ATTRv amyloidosis from non-endemic areas, manifest weakness and loss of all sensory modalities without clinically significant autonomic symptoms [22]. A characteristic feature of cardiomyopathy resulting from amyloidosis is heart failure with preserved ejection fraction on echocardiography [23,24]. Cardiac conduction abnormalities also frequently occur [4,20] (Table 1).

## 3. Ultrastructure of Tissue Damage

### 3.1. Atrophy and Degeneration Induced by Amyloid Fibrils

**Figure 1:**
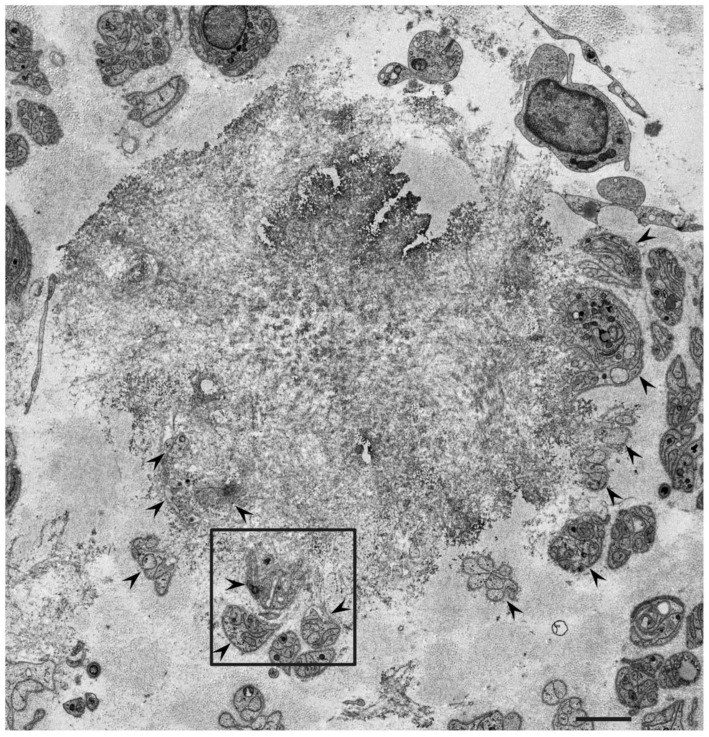
Representative electron microscopy photograph of Schwann cells near a mass of amyloid fibrils. A cross-section of the sural nerve biopsy specimen from a patient with ATTRv amyloidosis. Subunits of Schwann cells indicated by arrowheads are located in the periphery of a mass of amyloid fibrils [28]. A high-powered view in the box is shown in Figure 2. Uranyl acetate and lead citrate stain. Scale bar = 2 μm.

**Figure 2:**
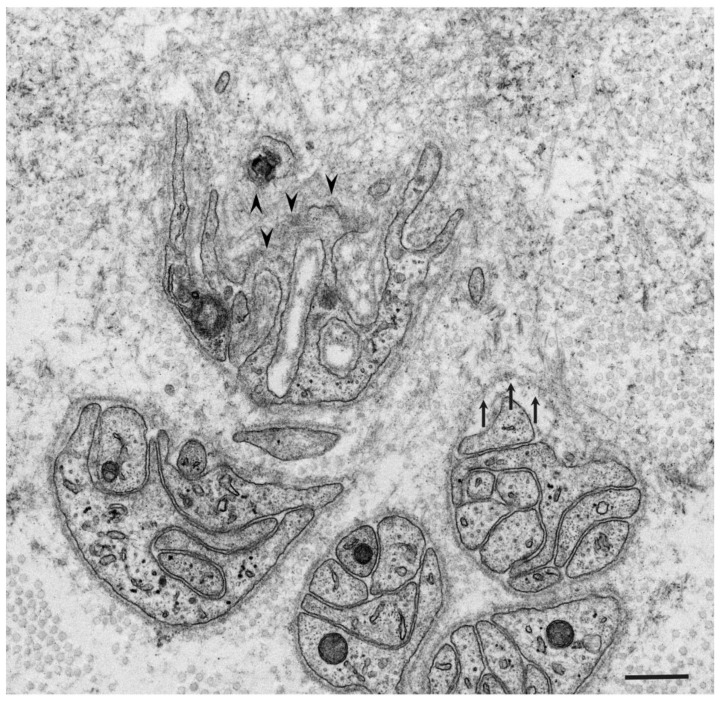
Atrophy of Schwann cells near amyloid fibrils. A cross-section of the sural nerve biopsy specimen from a patient with ATTRv amyloidosis. Basement and cytoplasmic membranes indicated by arrowheads are indistinct [28,29]. A basement membrane indicated by arrows seems to be pulled toward the center of an amyloid fibril mass [29]. Uranyl acetate and lead citrate stain. Scale bar = 0.5 μm.

**Figure 3:**
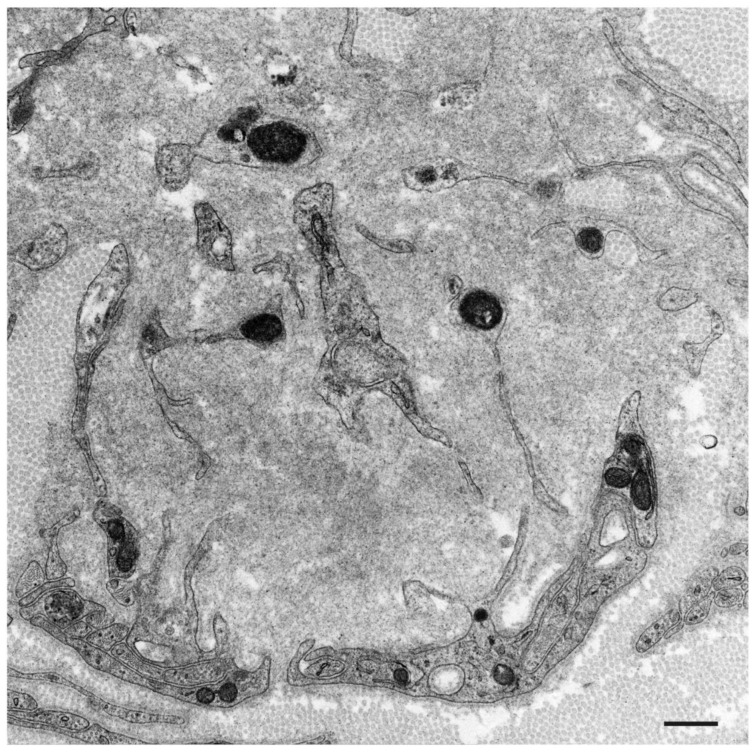
Subunits of Schwann cells surrounded by amyloid fibrils in AL amyloidosis. A cross-section of the sural nerve biopsy specimen. Schwann cells become atrophic and their basement and cytoplasmic membranes are indistinct [21]. Uranyl acetate and lead citrate stain. Scale bar = 0.5 μm.

According to studies of autopsy specimens obtained from patients with ATTRv amyloidosis, the extent of the degeneration of neurons in the sensory and autonomic ganglia seems to be positively correlated with the amount of amyloid, suggesting that neurodegeneration occurs as a result of amyloid deposition [4,25]. Electron microscopy examination of nerve biopsy specimens from ATTRv amyloidosis patients demonstrated atrophy of Schwann cells opposed to amyloid fibril aggregates and loss of axons associated with these Schwann cells (Figure 1 and Figure 2) [26,27,28,29]. The atrophy of Schwann cells near amyloid fibril masses has also been reported in specimens from AL amyloidosis patients (Figure 3) [21,30,31]. Similar findings have also been reported in the hearts of these patients [4].

The mechanisms of atrophy and subsequent degeneration of tissues near amyloid fibrils have not been fully elucidated. One of the possibilities is the stress or toxicity that is attributable to the amyloid fibrils themselves. A previous electron microscope study of nerve biopsy specimens from Portuguese ATTRv amyloidosis patients showed a long amyloid fibril penetrating the cytoplasmic membrane of a Schwann cell, suggesting direct damage of Schwann cells by amyloid fibrils [26]. In contrast, later studies did not describe this finding, but instead demonstrated distorted Schwann cells toward the direction of amyloid fibril elongation, suggesting mechanical stress resulting from traction [29]. The atrophy of Schwann cells tends to be more conspicuous in early-onset ATTRv amyloidosis patients from the conventional endemic foci in Portugal and Japan than in ATTRv amyloidosis patients from non-endemic areas [28,29]. This might be related to the size of the amyloid fibrils; the early-onset patients from the endemic foci have long and thick amyloid fibrils, whereas most patients from non-endemic areas have short and fine amyloid fibrils [28,29,32,33]. Mechanical stress during the process of amyloid fibril elongation may, in part, affect Schwann cells because these cells seem to be distorted toward the direction of amyloid fibril elongation (Figure 2) [29]. Similarly, autopsy specimens of the heart from early-onset ATTRv amyloidosis patients from conventional endemic foci revealed atrophy and degeneration of myocardial cells surrounded by long and thick amyloid fibrils, whereas short and fine amyloid fibrils and less conspicuous atrophy of myocardial cells were observed in patients from non-endemic areas [4].

Another possibility of the mechanisms of tissue damage is the toxicity of non-fibrillar states of amyloidogenic proteins [9,34,35,36]. In particular, many studies suggest the importance of oligomeric species, rather than mature amyloid fibrils, for tissue damage in Alzheimer’s disease and prion diseases [34,35]. Experiments using schwannoma or neuroblastoma cell lines indeed demonstrated the toxic effects of non-fibrillar TTR, thus supporting this view [37,38,39,40]. Studies using *Caenorhabditis elegans* or *Drosophila* also demonstrated the neurotoxicity of TTR in the absence of fibrillar amyloid deposition [41]. The extent of nerve fiber loss is more conspicuous, despite the smaller number of amyloid deposits, in late-onset ATTRv amyloidosis patients from non-endemic areas than in early-onset ATTRv amyloidosis patients from endemic foci [4], suggesting that the toxicity of non-fibrillar TTR participates in the mechanisms of neurodegeneration, especially in late-onset ATTRv amyloidosis patients from non-endemic areas.

### 3.2. Obscuration of Basement and Cytoplasmic Membranes

Another important finding regarding tissue damage resulting from amyloid deposition is the obscuration of the basement and cytoplasmic membranes near amyloid fibrils. Electron microscope studies of nerve biopsy specimens have demonstrated that the membranes of Schwann cells become obscure when they are near amyloid fibrils in both ATTR amyloidosis and AL amyloidosis patients (Figure 2 and Figure 3) [21,28,29,30,31]. The contours of Schwann cells may completely disappear when Schwan cells are surrounded by amyloid fibrils, suggesting that the destruction of these membranes occurs as a result of amyloid fibril formation [29]. Scattered cytoplasmic organelles may be found within the aggregates of amyloid fibrils as remnants of Schwann cells in such cases [29]. This destruction of Schwann cell membranes may play an important role in the demyelination reported in ATTRv amyloidosis patients [29,42]. Obscuration of basement and cytoplasmic membranes of cells constituting vessel walls, such as endothelial cells and pericytes, near amyloid fibrils has also been demonstrated in ATTRv amyloidosis patients [28].

A previous study of cardiac amyloid deposits suggested that TTR aggregation into fibrillar structures tends to occur in association with the basement membrane because the expression of basement membrane components, such as collagen IV, laminin, and fibronectin, increases in parallel with the accumulation of amyloid fibrils [43]. Studies of nerve biopsy specimens also revealed that amyloid fibrils are frequently found at or around basement membranes surrounding endoneurial microvessels or Schwann cells [6]. These findings suggest an affinity of the components constituting the basement membrane to those of amyloid fibrils. Additionally, a previous study suggested that cholesterol and anionic phospholipids might be important for TTR aggregation and TTR-induced cytotoxicity [44].

## 4. Insights into Therapeutic Strategies

The possible major therapeutic strategies for amyloidosis consist of the following: (1) reducing or preventing the production of causative proteins; (2) preventing the causative proteins from participating in the process of amyloid fibril formation; and/or (3) eliminating the already-deposited amyloid fibrils. As described earlier, the deposition of amyloid fibrils or non-fibrillar oligomers induces atrophy and subsequent degeneration of neighboring cells in amyloidosis. Therefore, intervention during the early stages of the disease process, before the occurrence of tissue damage by amyloid fibrils, seems to be an efficient approach.

From this viewpoint, the development of therapeutic agents to reduce or prevent the production of causative proteins is remarkable in both ATTR and AL amyloidoses. Liver transplantation was established as a treatment for ATTRv amyloidosis in the 1990s to prevent the production of the variant TTR from the liver [45]. Recently, patisiran, a short interfering RNA, and inotersen, an antisense oligonucleotide, were shown to reduce the production of TTR and have become available for ATTRv amyloidosis patients [46,47]. As these gene-silencing agents can prevent the production of both the variant and wild-type TTRs, these drugs are expected to be efficacious even for ATTRwt amyloidosis [48,49]. The main therapeutic strategy for AL amyloidosis is composed of chemotherapy against plasma cell dyscrasia to reduce or prevent the production of immunoglobulin light chains from clonally proliferated plasma cell or B cell lineage [10]. Although a combination of melphalan and steroids is the conventional chemotherapy for treating AL amyloidosis, other agents that are considered include melphalan, thalidomide, lenalidomide, pomalidomide, bortezomib, ixazomib, and daratumumab [10].

As for the strategy of preventing the causative proteins from participating in the process of amyloid fibril formation, TTR stabilizers, such as tafamidis and diflunisal, were demonstrated to be efficacious in patients with ATTR amyloidosis [50,51]. These drugs prevent the dissociation of TTR tetramers, which are physiologically stable, into unstable monomers, thereby inhibiting the subsequent misfolding and aggregation of TTR [6]. Tafamidis, in particular, is now available for not only ATTRv amyloidosis but also ATTRwt amyloidosis patients who have cardiomyopathy [8,52]. The use of doxycycline, a derivative of tetracycline that inhibits matrix metalloproteinases dysregulated in tissues in patients with ATTR and AL amyloidoses [53,54], is considered another future add-on option to inhibit amyloid fibril formation [55,56,57].

Regarding the elimination of amyloid fibrils, monoclonal antibodies against the components of amyloid deposits have been considered therapeutic agent candidates. A humanized IgG1 monoclonal antibody against serum amyloid P component (SAP), which is a plasma glycoprotein found in any type of amyloid deposit, was considered a therapeutic agent for systemic amyloidosis along with the use of an agent that depletes circulating SAP [58]. Although a phase 1 clinical trial demonstrated the shrinkage of amyloid deposits [59], the development of this antibody has been discontinued [18]. Several monoclonal antibodies specific to TTR or immunoglobulin light chain have also been developed [60,61,62,63,64]. Among these, phase 3 clinical trials of an IgG1 monoclonal antibody against the kappa and lambda light chain amyloid fibrils (CAEL-101) are ongoing (NCT04512235 and NCT04504825) [7].

## 5. Conclusions

Amyloidosis is a group of diseases in which amyloid fibrils are deposited in various organs, including the nervous system, heart, lungs, liver, kidneys, gastrointestinal tract, and soft tissues. Amyloid deposits may be localized to a single organ, as in the case of Alzheimer’s disease, or systemic, such as in ATTR and AL amyloidoses.

According to studies of autopsy specimens obtained from patients with ATTRv amyloidosis, the extent of the degeneration of neurons in the sensory and autonomic ganglia is positively correlated with the amount of amyloid, suggesting that neurodegeneration occurs as a result of amyloid deposition [4,25]. Electron microscopy examination of nerve biopsy specimens from ATTRv amyloidosis and AL amyloidosis patients demonstrated atrophy of the Schwann cells and loss of associated axons near amyloid fibril aggregates [21,26,27,28,29,30,31]. Similar findings also were reported in cardiac autopsy specimens from ATTR amyloidosis patients [4]. The mechanisms of atrophy and subsequent degeneration of tissues neighboring amyloid fibrils have not been fully elucidated. In addition to the stress or toxicity attributable to amyloid fibrils themselves, the toxicity of the non-fibrillar states of amyloidogenic proteins may also participate in the mechanisms of tissue damage [9,34,35,36]. In particular, many studies suggest the importance of non-fibrillar oligomeric species, rather than mature amyloid fibrils, for tissue damage in Alzheimer’s disease and prion diseases [34,35].

Another important finding of tissue damage resulting from amyloid deposition is the obscuration of the basement and cytoplasmic membranes of Schwann cells and cells constituting endoneurial microvessels near amyloid fibrils [28,29]. As amyloid fibrils were frequently found at or around basement membranes surrounding endoneurial microvessels or Schwann cells [6], an affinity of components constituting the basement membrane to those of amyloid fibrils may also play an important role in tissue-damage associated with amyloidosis.

The possible major therapeutic strategies for amyloidosis consist of the following: (1) reducing or preventing the production of causative proteins; (2) preventing the causative proteins from participating in the process of amyloid fibril formation; and/or (3) eliminating already-deposited amyloid fibrils. In addition to liver transplantation to prevent the production of variant TTR [45], patisiran, a short interfering RNA, and inotersen, an antisense oligonucleotide, can reduce the production of TTR and have become available for ATTRv amyloidosis patients [46,47]. As these gene-silencing agents can prevent the production of both variant and wild-type TTRs, these drugs are also expected to be efficacious even for ATTRwt amyloidosis [48,49]. The main therapeutic strategy for AL amyloidosis is composed of chemotherapy against plasma cell dyscrasia [10]. As for the strategy of preventing the causative proteins from participating in the process of amyloid fibril formation, TTR stabilizers such as tafamidis and diflunisal have been demonstrated to be efficacious in patients with ATTR amyloidosis [50,51]. In particular, tafamidis is now available for not only ATTRv amyloidosis but also for ATTRwt amyloidosis patients who have cardiomyopathy [8,52]. Future therapeutic options include doxycycline as an add-on agent to prevent amyloid fibril formation, and monoclonal antibodies against SAP, TTR, and immunoglobulin light chains to remove the already-deposited amyloid fibrils or their components [18,55,56,57,60,61,62,63,64]. As the development of these novel disease-modifying therapies is remarkable, early diagnosis and appropriate selection of treatment is becoming more and more important for patients with amyloidosis.

## Figures and Tables

**Table 1:** Characteristics of ATTR and AL amyloidoses.

	ATTR Amyloidosis		AL Amyloidosis
	ATTRwt Amyloidosis	ATTRv Amyloidosis	
Precursor protein	Transthyretin	Transthyretin	Immunoglobulin light chain
Acquired or hereditary	Acquired	Hereditary	Acquired
Underlying condition	Aging	Mutation in the *TTR* gene	Plasma cell dyscrasia
Major organ involvement	Heart	Heart	Heart
	Tendon/ligament	Peripheral nervous system	Peripheral nervous system
		Gastrointestinal tract	Gastrointestinal tract
		Eye	Kidney
			Liver
			Soft tissue

ATTRv amyloidosis = hereditary ATTR amyloidosis; ATTRwt amyloidosis = wild-type ATTR amyloidosis.

## Data Availability

Not applicable.
